# Comparative genomics and phylogenetic analysis of six Malvaceae species based on chloroplast genomes

**DOI:** 10.1186/s12870-024-05974-w

**Published:** 2024-12-26

**Authors:** Yiwang Zhong, Beibei Bai, Yangyang Sun, Ke Wen, Yang Qiao, Lijun Guo, Huidong Deng, Yingjun Ye, Liying Feng, Xuejie Feng

**Affiliations:** 1 Sanya Institute, Hainan Academy of Agricultural Sciences, Sanya, 572024 China; 2 Institute of Tropical Fruit Trees, Hainan Academy of Agricultural Sciences, Haikou, 571100 China; 3 Key Laboratory of Genetic Resources Evaluation and Utilization of Tropical Fruits and Vegetables (Co-construction by Ministry and Province), Ministry of Agriculture and Rural Affairs, Haikou, 571100 China; 4 Key Laboratory of Tropical Fruit Tree Biology of Hainan Province, Haikou, 571100 China; 5 Yazhouwan National Laboratory, Sanya, 572024 China

**Keywords:** Chloroplast genome, Malvaceae, Sequence characteristic, Comparative analysis, Phylogeny

## Abstract

The Malvaceae family, comprising 9 subfamilies and 4,225 species, includes economically significant taxa, such as *Ceiba pentandra*, *Gossypium ekmanianum*, *Gossypium stephensii*, *Kokia drynarioides*, *Talipariti hamabo*, and *Durio zibethinus*. Chloroplast (cp) genome research is crucial for elucidating the evolutionary divergence and species identification within this family. In this study, we assembled and annotated cp genomes of six Malvaceae species, conducting comprehensive comparative genomic and phylogenomic analyses. The assembled genomes range from 160,495 to 163,970 bp in size, with 125–129 genes annotated. Notable differences were observed in the IR (inverted repeat) regions, and SSR analysis revealed that *Durio zibethinus* has the highest number of specific variation sites. Among the six species, *Talipariti hamabo* uniquely exhibits more palindromic repeats than forward repeats. Seven highly mutated regions were identified, offering potential markers for species identification. Phylogenetic reconstruction using the maximum likelihood method revealed two primary clades within Malvaceae: Byttneriina and Malvadendrina. Within Malvadendrina, the subfamily Helicteroideae represents the earliest divergence, followed by Sterculioideae. This study provides a robust phylogenetic framework and valuable insights into the classification and evolutionary history of Malvaceae species.

**Supplementary Information:**

The online version contains supplementary material available at 10.1186/s12870-024-05974-w.

## Introduction

Chloroplasts (cp), vital organelles for photosynthesis, are ubiquitously found in plants and eukaryotic algae [1, 2]. With the advent of the genomics era, significant progress has been made in the study of cp genomes [3]. Despite its relatively compact size, the cp genome demonstrates a remarkable balance between conservation and variability in both gene content and structural organization across diverse plant species [3, 4]. The cp genome typically exhibits a quadripartite structure, comprising a large single-copy (LSC) region, a small single-copy (SSC) region, and two inverted repeat (IR) regions. This structure encodes between 110 and 130 genes, with genome sizes ranging from 110 to 240 kb [5, 6]. Compared to the mitochondrial and nuclear genomes, the cp genome is generally inherited and does not undergo recombination [7]. Although the structure of the cp genome is generally conserved in angiosperms, significant variations have been observed in genome size, structural organization, and gene substitution rates [8, 9].

Owing to its mode of maternal inheritance, the cp genome offers direct insights into the evolutionary history of maternal lineages in plants [10, 11]. Furthermore, the conserved gene sequences and structural characteristics of the cp genome make it an ideal molecular marker for investigating large-scale phylogenetic relationships among plants [12, 13]. The evolution of the cp genome also reflects the history of plant adaptation to environmental changes [14]. The loss, rearrangement, and horizontal transfer of certain genes reveal the mechanisms by which plants have adapted to different ecological environments throughout their evolutionary history [15, 16]. This information is pivotal for elucidating plant diversity, evolutionary mechanisms, and ecological adaptability. Consequently, the investigation of the cp genome not only deepens our understanding of photosynthesis and cellular functions in plants but also supplies critical molecular evidence for studies on plant evolution, biodiversity, and ecological adaptation [17, 18].

Malvaceae family, also known as the mallow family, comprises approximately 244 genera and around 4,225 species, including well-known plants such as okra, cotton, cacao, roses, and durian [19–21]. The species of the Malvaceae family are widely distributed across tropical, subtropical, and temperate regions of the world. They are found in diverse habitats, ranging from forests and grasslands to arid and coastal areas [22]. The Malvaceae family is distinguished by its extensive variety of species, ranging from diminutive herbaceous plants to towering trees. And they are of great ornamental, economic, edible and medicinal importance. Such as the flowers, typically exhibiting five petals and vibrant hues, are a hallmark of the family and are frequently cultivated in gardens and landscape design [23–25]. Additionally, such as cotton, which is a major source of natural fiber [26], Durian is renowned as the “King of Fruits” due to its distinctive aroma and creamy texture [27]. *Ceiba pentandra*, native to tropical America, is widely cultivated across tropical regions worldwide. It is extensively distributed in the tropical areas of Asia and Africa, with particularly abundant populations in Indonesia [28]. Some species of the Malvaceae family have been used in traditional medicine for centuries. Known for its anti-inflammatory, antioxidant, and liver-protective properties, *Malva sylvestris* is used to treat respiratory diseases, skin inflammation, and gastrointestinal problems [29]. The fibers from Velvetleaf (*Abutilon theophrasti*) possess excellent strength and durability, and they are commonly used for making ropes and fabrics [30]. In molecular biology research, plants of the Malvaceae family, due to their diversity and wide distribution, have become important models for studying plant evolution, genomics, and ecology [23]. With advancements in genomics and bioinformatics technologies, research on the phylogeny, gene functions, and metabolic pathways of Malvaceae plants will continue to deepen, enhancing our understanding and application of plant biology.

In the past few decades, phylogenetic research on the Malvaceae family has made significant progress [21, 23]. Traditional morphological classification methods have gradually been supplemented and replaced by molecular systematics studies, which primarily utilize sequence data from nuclear genes, cp genes, and mitochondrial genes [31–33]. The Malvaceae family is divided into nine subfamilies: Byttnerioideae, Grewioideae, Helicteroideae, Sterculioideae, Brownlowioideae, Dombeyoideae, Tilioideae, Bombacoideae, and Malvoideae [19]. Studies based on DNA fragments have found that Bombacoideae and Malvoideae form a sister clade, while Byttnerioideae and Grewioideae form a sister clade with the remaining subfamilies [34–37]. Studies based on cp genomes suggest that Byttnerioideae and Grewioideae form a distinct clade, while the remaining seven subfamilies form another clade [38–40]. Currently, the datasets cover relatively few species within each subfamily. Therefore, expanding the species-level sampling of Malvaceae and conducting phylogenetic studies based on cp genomes can better clarify the evolutionary relationships within the family.

In this study, we reported the cp genomes of six Malvaceae species, six species have had their nuclear genomes published, with species identification already confirmed in these publications [25, 41–44]. Although chloroplast genomes of several durian cultivars have been published [45–47], the complete chloroplast genome data for the ‘Monthong’ cultivar remain unclear. Similarly, the chloroplast genome data for the other five species also lack consistency or completeness. A comparative analysis was conducted across these six species, elucidating structural variations in each. Additionally, leveraging cp genome data, we identified the hotspot regions of variation in the cp genome and elucidated the phylogenetic relationships among the nine subfamilies of the Malvaceae. This study will provide valuable insights for clarifying species identification and the phylogenetic history of Malvaceae plants.

## Materials and methods

### Plant material sampling and DNA extraction

The data for the five species used in this study were downloaded from National Center for Biotechnology Information (https://www.ncbi.nlm.nih.gov/) on 10 January 2024. One species was sequenced from our own sample collection. The data for *Ceiba pentandra* (PRJNA905654) was obtained from the Institute of Cotton Research Yacheng Base, Chinese Academy of Agricultural Sciences, Sanya, Hainan Province, China (18°23′43.3896″N, 109°10′27.4620″E). Samples of *Gossypium ekmanianum* and *Gossypium stephensii* (PRJNA739494) were sourced from the National Wild Cotton Nursery in Sanya, China, managed by the Institute of Cotton Research, Chinese Academy of Agricultural Sciences (ICR-CAAS). Fresh leaf tissue from *Kokia drynarioides* (PRJNA400144) was collected from the Iowa State University greenhouse. *Talipariti hamabo* (PRJNA759075) tissue was obtained from the Institute of Botany, Jiangsu Province, Chinese Academy of Sciences. The *Durio zibethinus* samples were collected from the Yucai durian plantation in Sanya, Hainan. For *D. zibethinus*, young, developing leaf tissues from healthy plants were immediately frozen in liquid nitrogen and stored at -80 °C to preserve DNA integrity. Total genomic DNA was extracted using the CTAB method [48]. The genome was sequenced on the Illumina NovaSeq 6000 platform using next-generation sequencing technology, with paired-end reads of 150 bp and an insert size of 350 bp. Genome indexing and alignment were performed using Bowtie2 V2.4.1 [49] and SAMtools V1.13 [50], coverage and depth calculations were conducted using the bamdst V1.1.0 (https://github.com/shiquan/bamdst).

### Characterization of cp genomes in six Malvaceae species

The sequencing data were subjected to quality control and filtering using Fastp V0.15.0 [51]. After filtering and screening the sequencing data, the cp genome was automatically assembled using GetOrganelle V1.7.1 [8] with k-mer parameters set to 21, 45, 75, 85, and 105. The assembly was visualized using Bandage V0.8.1 [52], which facilitated the removal of redundant contigs and the circularization of the sequence. The assembled cp genome was annotated using CPGAVAS2 [53], and a circular map was generated using OGDRAW [54].

### Simple repeat sequence annotation

Simple sequence repeats (SSRs), including mononucleotide, dinucleotide, trinucleotide, tetranucleotide, pentanucleotide, and hexanucleotide SSRs, were predicted using MISA [55]. The parameters were set to > 10 repeat units for mononucleotide SSRs, > 6 repeat units for dinucleotide SSRs, ≥ 5 repeat units for trinucleotide SSRs, 24 repeat units for tetranucleotide SSRs, and ≥ 3 repeat units for both pentanucleotide and hexanucleotide SSRs.

### Characterization and comparative analysis of genomes

The GC content of each cp genome, including the LSC, SSC, and IR regions, was determined using Extractseq V6.6.0 and Seqkit V2.3.1. The number of genes in the total cp genome, tRNA, and rRNA was determined from the GB files of each VP genome. Repetitive sequences, including forward (F), reverse (R), complement (C), and palindromic (P) repeats, were identified using REPuter [56] with a minimum repeat size set to 30 bp and a Hamming distance of 3. Gene loss was visualized using the heatmap function from the R package. The cp genome sequences were compared using the mVISTA program [57]. IRscope V0.1 [58] was used to visualize the contraction and expansion of the IR boundaries between the four regions of the genome (LSC/IRb/SSC/IRa). Sequence alignment was performed using MAFFT V7.310 [59], and a sliding window analysis was conducted with DnaSP V6.12.03 [60] to calculate Pi across the cp genome. The step size was set to 200 bp, with a window length of 600 bp.

### Phylogenetic analysis

To elucidate the phylogenetic relationships within the Malvaceae family, 141 cp genome sequences of Malvaceae were downloaded from the NCBI database (Table S2). Using *Dipterocarpus turbinatus* (NC_046842.1) as an outgroup, a phylogenetic analysis was conducted on a total of 148 cp genomes, including six species assembled in this study. We first corrected the SSC regions of all cp genomes by reversing them. After aligning the sequences consistently for all species, we constructed the phylogenetic tree. Multiple sequence alignments were performed using MAFFT V7.310 [59], were trimmed using TrimAl V1.4 [61] with a minimum conservation threshold and a gap threshold of 20%. Phylogenetic reconstruction was conducted using the Maximum Likelihood (ML) method. The ML tree was generated with 1000 bootstrap replicates using the GTRGAMMA model, implemented in RAxML V8.1.17 [62]. The analyses were run with the command “raxmlHPC-PTHREADS-SSE3 -f a -x 12345 -p 12345 -s all.fasta -m GTRGAMMA -N 1000 -n output”. We downloaded the protein files of the nuclear genomes for 22 species and identified single-copy orthologous genes and constructed species tree using OrthoFinder V3.0 [63]. iTOL (https://itol.embl.de/) was used to enhance and annotate the evolutionary tree, as well as to incorporate subgroup information.

## Result

### Characterization of cp genomes in six Malvaceae species

**Fig. 1:**
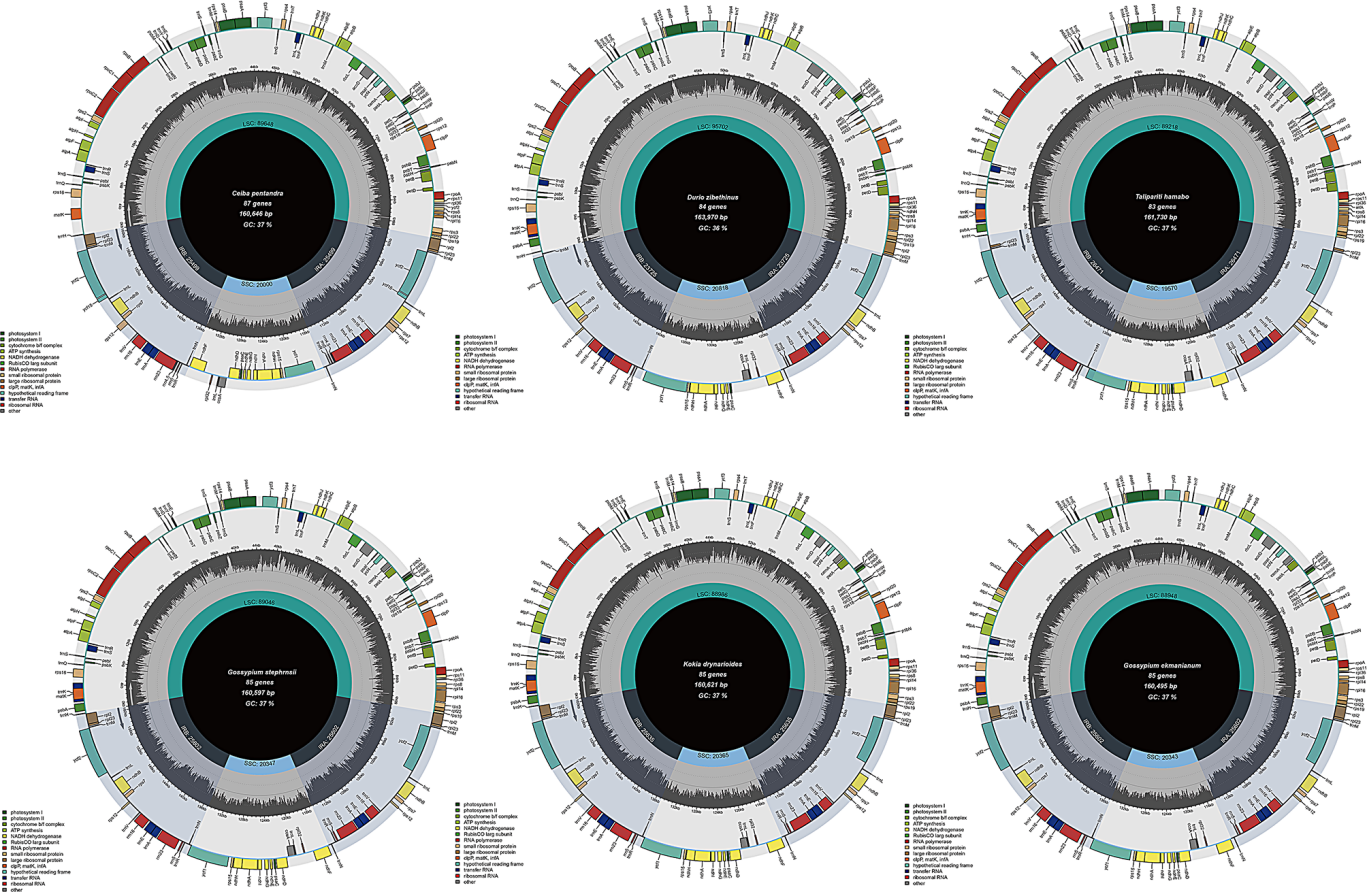
Cp genome maps of six Malvaceae. Genes shown outside of the outer layer circle are transcribed clockwise, while those insides are transcribed counterclockwise. The genes belonging to different functional groups are color-coded. The dark gray area of the inner circle denotes

In this study, we assembled the cp genomes of six species within the Malvaceae family. These cp genomes exhibit the typical quadripartite structure, comprising a LSC region, a SSC region, and two IR regions (Fig. 1). The genome sizes range from 160,495 to 163,970 bp, GC content of 35.83-37.34%, and the number of genes identified varies between 125 and 129 (Table 1). The GC content of the IR regions is significantly higher than that of the LSC and SSC regions. This pattern of GC content distribution is consistent with observations in other plant species [14, 64]. Among the six species, *T. hamabo* has the fewest protein-coding genes, with a total of 83. *D. zibethinus* contains the highest amount of rRNA with 8. The content of tRNA (36) is consistent across all species.

**Table 1:** Comparison of cp features among six Malvaceae plants

	*C. pentandra*	*D. zibethinus*	*G. ekmanianum*	*G. stephensii*	*K. drynarioides*	*T. hamabo*
Total length (bp)	160,646	163,970	160,495	160,597	160,621	161,730
GC (%)	36.81	35.83	37.19	37.18	37.34	36.9
LSC.length (bp)	89,648	95,702	88,948	89,046	88,986	89,218
SSC.length (bp)	20,000	20,818	20,343	20,347	20,365	19,570
IR.length (bp)	50,998	47,450	51,204	51,204	51,270	52,942
Total number of genes	129	128	127	127	127	125
Protein-coding genes	87	84	85	85	85	83
tRNA genes	36	36	36	36	36	36
rRNA genes	6	8	6	6	6	6

### Codon bias and sequence repeats analysis

**Fig. 2:**
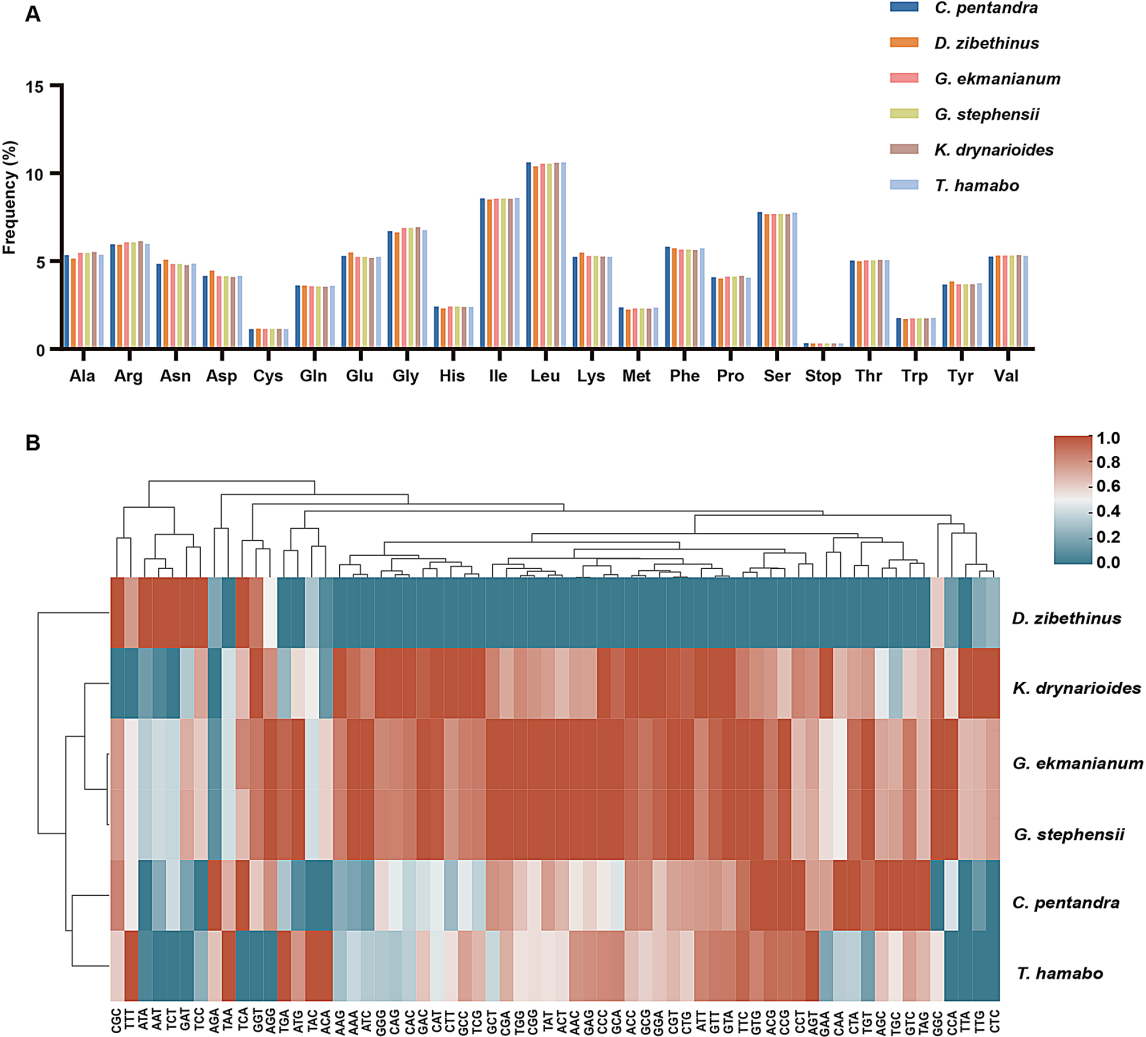
Analysis of codon preferences in the cp genomes of six Malvaceae species. **A**. amino acid usage; **B**. codon usage

The patterns of codon usage and nucleotide composition contribute to establishing a theoretical foundation for the genetic modification of cp genomes [65]. The codon usage frequency of protein-coding genes in the cp genomes of six assembled Malvaceae species was thoroughly analyzed. These genomes contain between 25,128 and 26,256 codons, with *D. zibethinus* having the fewest and *G. ekmanianum* and *G. stephensii* containing the most. Among all the codons, leucine (Leu) emerged as the most prevalent amino acid, with a frequency ranging from 10.25 to 10.40%. This was followed by isoleucine (Ile), with a frequency between 10.39% and 10.62%. Conversely, cysteine (Cys) was the least abundant, with a frequency of 1.13–1.15% (Fig. 2A, Table S3). After logarithmically transforming the usage counts of all amino acids, statistical analysis and clustering were performed. The results revealed differences in the usage of different amino acids encoded by the same codon. *D. zibethinus* exhibited significant differences compared to the other five species. Furthermore, the stop codon TAA was preferentially used (Fig. 2B, Table S4).

**Fig. 3:**
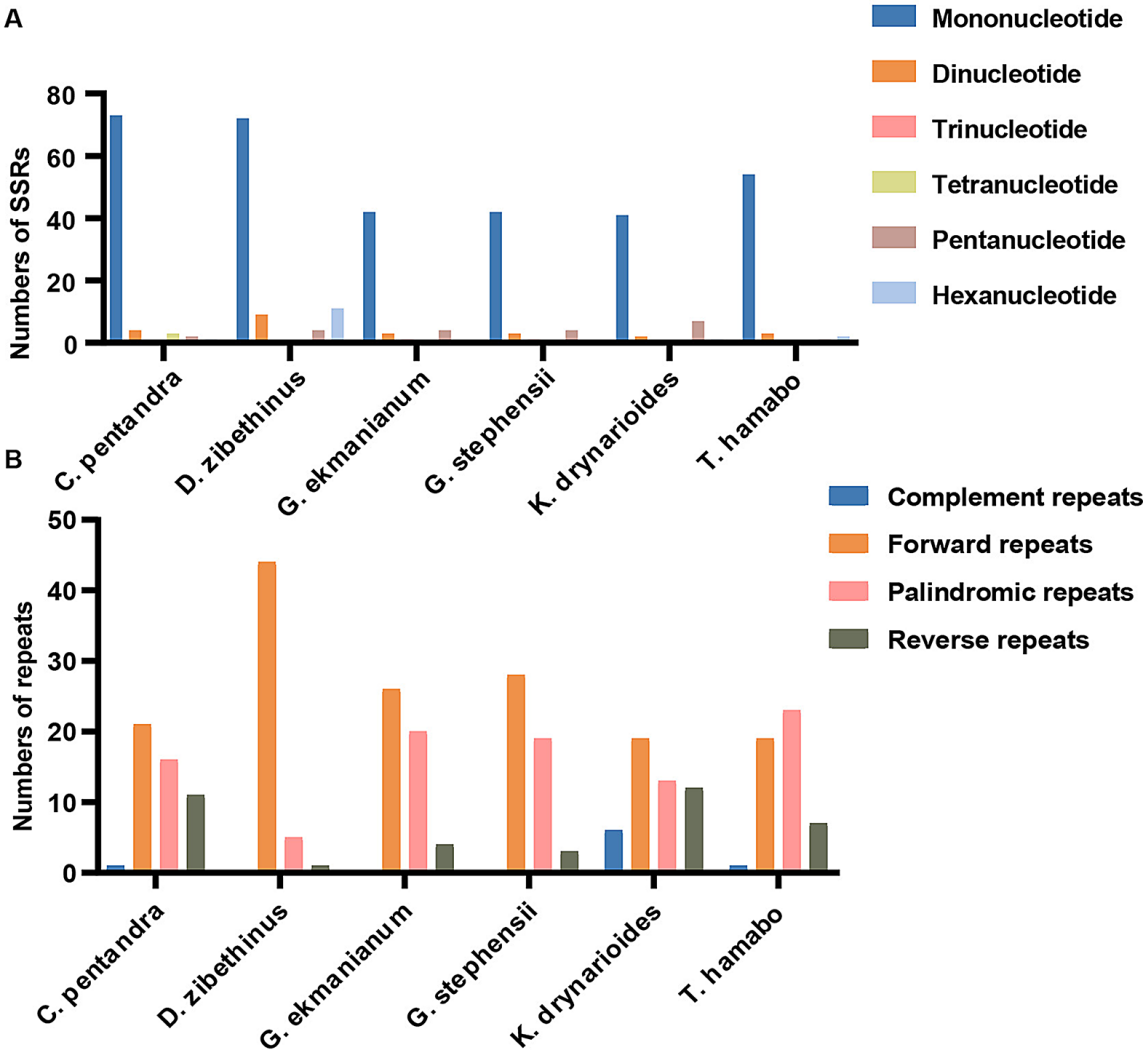
Analysis of sequence repeat in the six Malvaceae cp genomes. **A**. The type frequency of different SSR types. **B**. Frequency of identified long repeats types

We further conducted an analysis of SSR and repetitive sequences in six species. Upon statistical analysis of single nucleotide polymorphisms, we found that *C. pentandra* exhibited the highest number, while *K. drynarioides* had the lowest. Notably, except for *C. pentandra*, tetranucleotides were absent in the other five species. The overall SSR analysis revealed that *D. zibethinus* contained the highest number of SSRs (96) (Fig. 3A). Repetitive sequence analysis indicated that *D. zibethinus* had a significantly higher number of forward repeats compared to the other five species, whereas *K. drynarioides* exhibited the highest number of complement repeats (Fig. 3B).

### Comparative analysis between six Malvaceae species

**Fig. 4:**
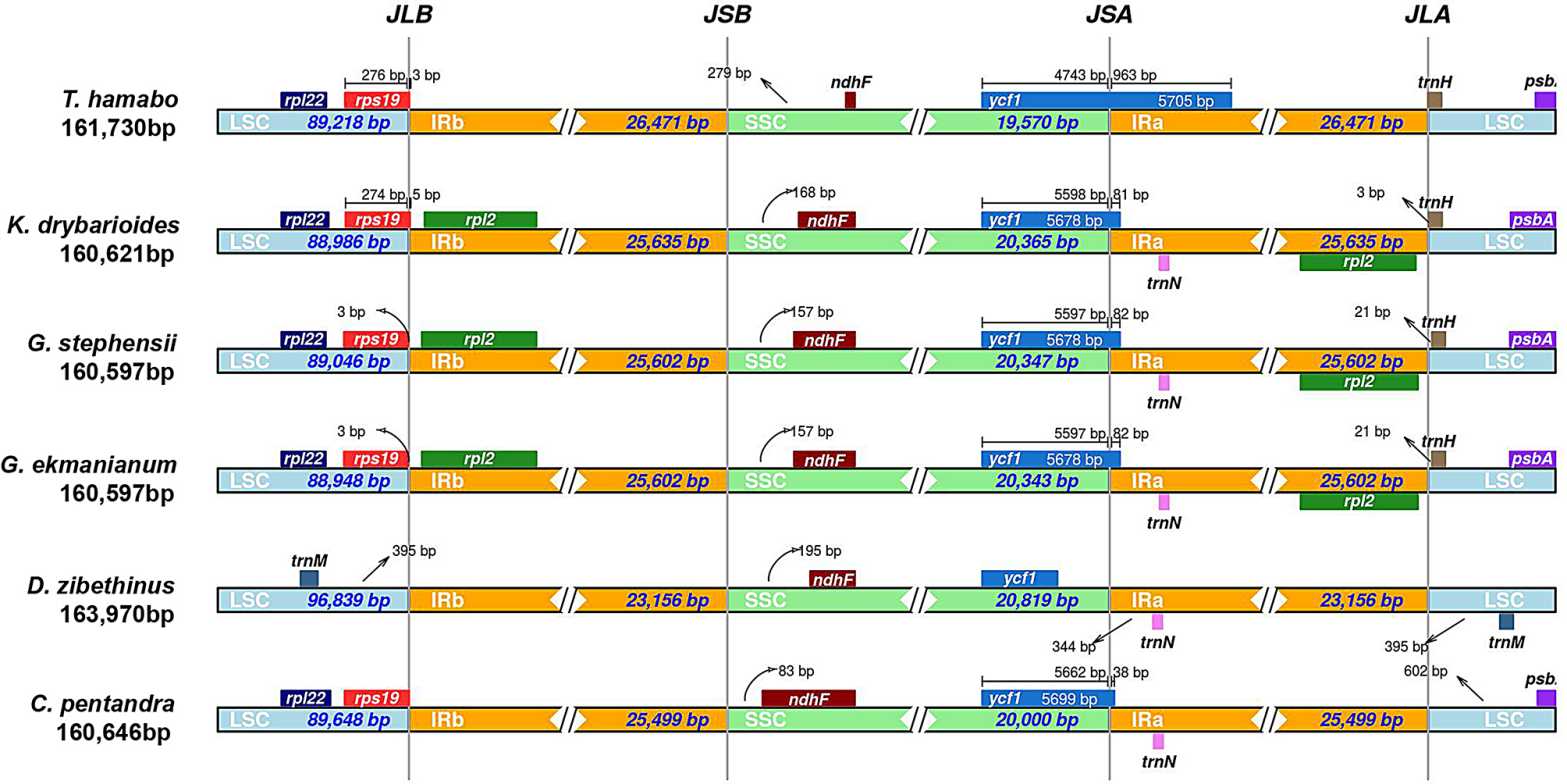
Comparison of the borders of the LSC, SSC, and IR regions among six Malvaceae cp genomes

The expansion and contraction of the IR region are major driving forces in the evolution of land plants [66–69]. In this study, we compared the adjacent genes and boundary conditions of the four regions (LSC-IRb-SSC-IRa) among six species (Fig. 4). The results revealed that the *rps19* gene is located at the LSC and IRb boundary, but this gene is absent in durian compared to the other five species. The *ndhF* gene is situated in the SSC region, with a significant reduction in the nucleotide sequence of the *ndhF* gene in *T. hamabo*. The *ycf1* gene spans the SSC and IRa boundary, with 963 bp of this gene located in the IRa region in *T. hamabo*, whereas in *D. zibethinus*, the entire gene is situated in the SSC region. In the other four species, the *ycf1* gene extends less than 100 bp into the IRa region. The genome size expansion in *D. zibethinus* is primarily observed in the LSC region. The *rpl2* gene is only present in *K. drynarioides*, *G. stephensii*, and *G. ekmanianum*. Additionally, the inverted repeat regions in *D. zibethinus* are the shortest compared to the other five species.

**Fig. 5:**
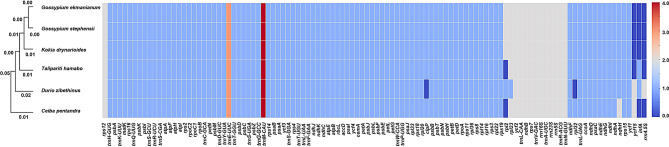
Phylogeny and gene loss in six Malvaceae species

Through phylogenetic analysis and gene loss statistics of six species, we found that *C. pentandra* diverged first, followed by *D. zibethinus*, then *T. hamabo*. The two species of the genus *Gossypium* form sister branches (Fig. 5). The gene loss statistics revealed that the gene with the highest frequency in the six species is *trnM-CAU* (4 copies), followed by *trnY-GUA* (3 copies). Additionally, we found that *D. zibethinus* exhibits gene loss in *clpP* and *rpl32*, and *rpl2* is lost in *T. hamabo* and *C. pentandra*. There is only one copy of *rpl2* in *D. zibethinus*, whereas the remaining three species have two copies. Both *D. zibethinus* and *C. pentandra* have two *ycf15* genes, while the other four species show a loss of this gene (Fig. 5; Table S5).

**Fig. 6:**
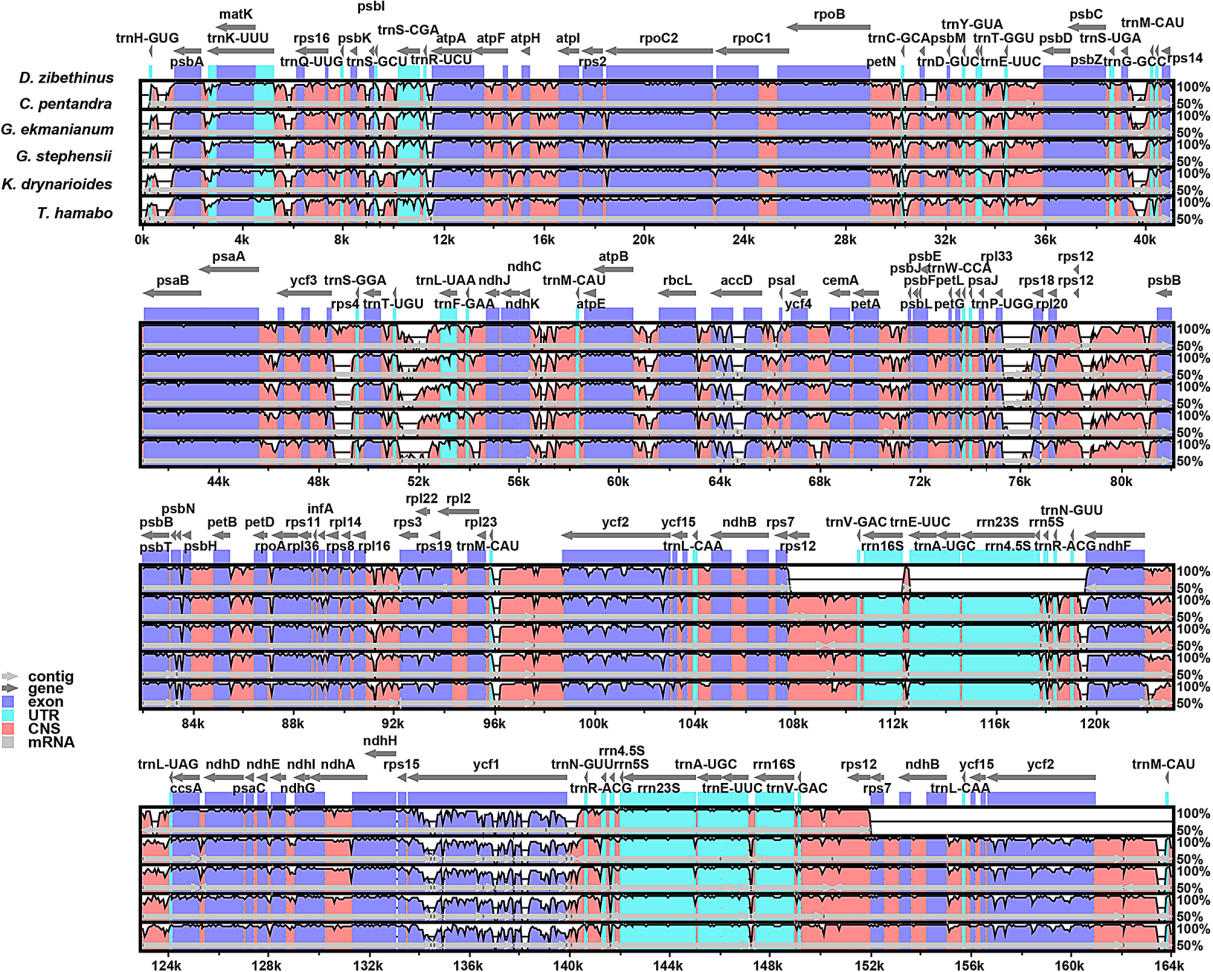
Analysis of sequence alignment of six Malvaceae cp genomes

We also analyzed the differences in the cp genomes of six species and further explored their genetic relationships (Fig. 6). Using mVISTA for global sequence alignment, with *D. zibethinus* sequence and annotation file as references, we observed variations across different regions among the six species. The results indicated that the cp genomes in Malvaceae species are highly conserved, with few variable regions, consistent with previous studies [66–68]. Additionally, we found that the IR regions are more conserved compared to the LSC and SSC regions, exhibiting fewer variable sites. Most variations were observed in the conserved non-coding sequences (NCS), with coding regions showing less divergence than non-coding regions. Among the coding genomes, genes with significant sequence differences included *rpl2*, *ycf2*, *ndhF*, and *ndhD*.

### Identification of variability hotspots

**Fig. 7:**
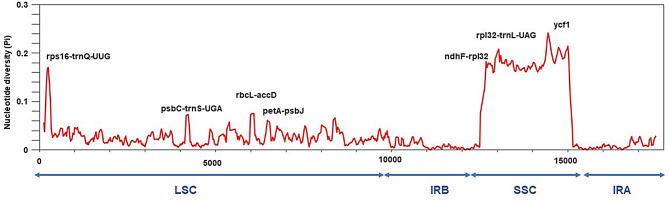
Nucleotide polymorphism of the cp genomes of six Malvaceae species

We performed whole cp genome analyses on six newly assembled species using DnaSP V6.12.03 [60], calculating Pi and Theta values (Fig. 7; Table S6). The mean Pi value of the aligned gene sequences was 0.03708, and the mean Theta value was 0.04431. We identified seven highly variable regions: *petA-pabJ*, *rbcL-accD*, *rpl32-trnL-UAG*, *rps16-trnQ-UUG*, *psbC-trnS-UGA*, *ndhF-rpl32*, and *ycf1*. Of these, four were located in the LSC region, while the remaining three were in the SSC region (Fig. 7). This pattern aligns with previous findings indicating that the IR region is typically more conserved than the LSC and SSC regions.

### Phylogenetic reconstruction of Malvaceae

**Fig. 8:**
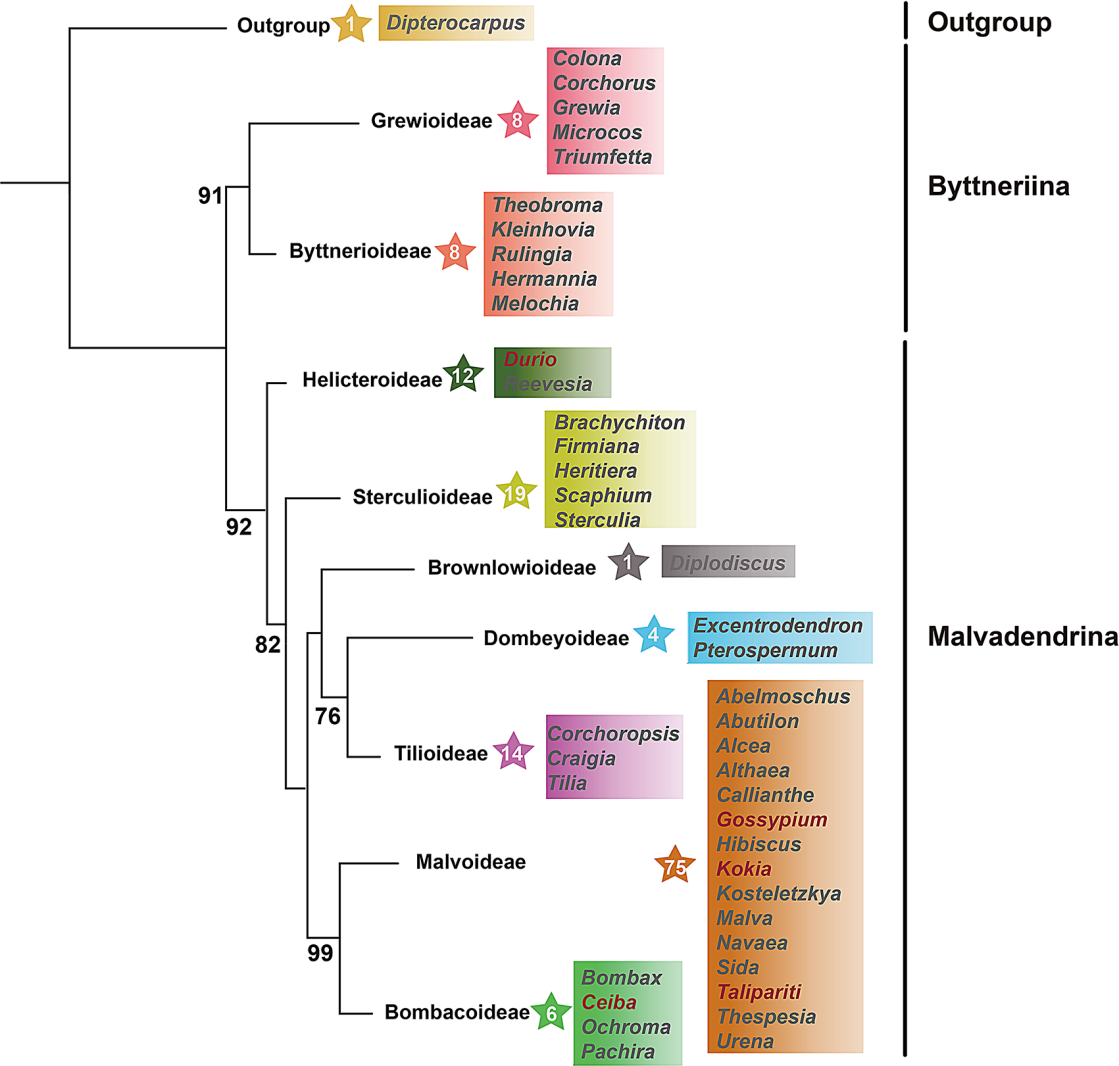
Phylogenetic relationships among subfamilies with complete cp sequence of Malvaceae. Species in genus assembled in this study are indicated in red text. The numbers within star symbols represent the number of species included

To elucidate the phylogenetic relationships within the Malvaceae family, we constructed a phylogenetic tree using the Maximum Likelihood (ML) method based on 148 genomes, including all genes and intergenic spacers (Fig. 8; Fig. S2). The results show that Byttnerioideae and Grewioideae form one clade, while the remaining seven subfamilies cluster into another clade. The phylogenetic relationships among the subfamilies Brownlowioideae, Tilioideae, and Dombeyoideae are closer. *K. drynarioides* is more closely related to *Thespesia populnea*. Genus *Talipariti* clusters together with *Hibiscus* and *Urena*, while *C. pentandra* forms a clade with the genus *Pachira*. *G. ekmanianum* and *G. stephensii* form a small clade. The topology of the phylogenetic tree has strong support at each node. All branches had support rates above 70%.

**Fig. 9:**
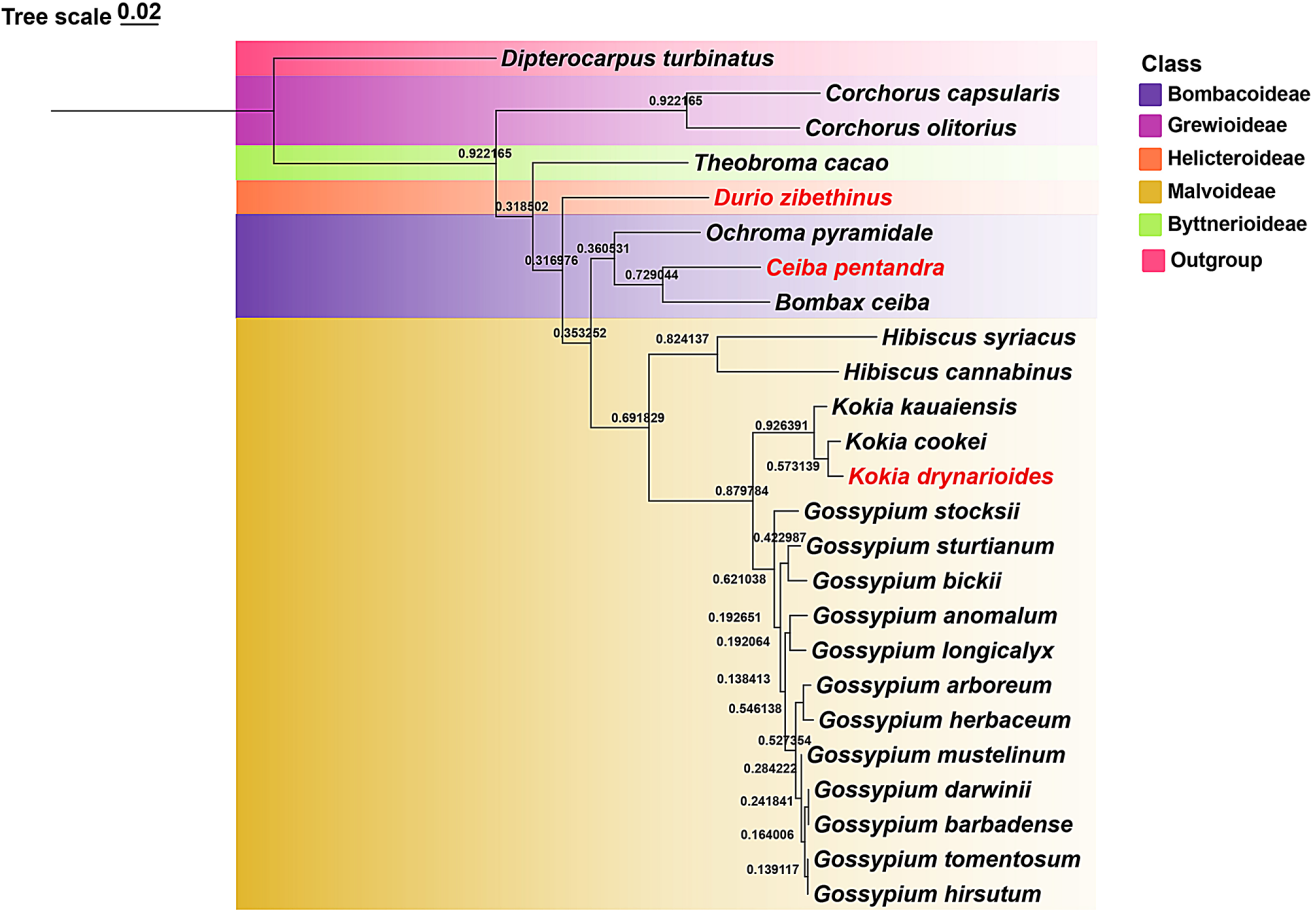
The phylogenetic relationships constructed based on homologous genes derived from the protein sequences of 25 nuclear genomes

To validate the accuracy of the phylogenetic tree, we selected the nuclear genomes of 24 Malvaceae species and one outgroup species that are currently available online. Using homologous genes, we reconstructed a species tree (Fig. 9; Table S7). Grewioideae diverged first as the basal clade, followed by Byttnerioideae.

## Discussion

### Chloroplast genome features in Malvaceae

The comparative analysis of plant genomes offers novel insights into patterns of molecular evolution cp sustain life on Earth by converting solar energy into carbohydrates through photosynthesis and oxygen release [70–72]. The advent of next-generation sequencing (NGS) technologies has equipped scientists with more rapid and cost-efficient methods for sequencing cp genomes [73, 74]. These advancements have significantly accelerated genomic research, enabling more comprehensive and detailed analyses of cp genome. This study utilized NGS data to obtain a detailed picture of the cp genome structures of six species within the Malvaceae. The GC content varies across the LSC, IR, and SSC regions, with the IR region having the highest GC content. The higher GC content in the IR region may be attributed to the abundance of rRNA genes in this region [75, 76]. Angiosperms exhibit a conserved quadripartite structure comprising two IR regions, a LSC region, and a SSC region, with genome sizes ranging from 110 kb to 240 kb [77–79]. The cp genomes of the six Malvaceae species we assembled range from 160,495 to 163,970 bp (Fig. 1), GC content ranges between 35.83% and 37.34%, indicating the highly conserved nature of cp. genomes in Malvaceae species.

In the structure of cp genomes, the IR region is typically the most conserved. Due to the highly conserved nature of cp. genomes, gene loss within this organelle provides crucial insights into species evolution [80]. Gene loss primarily occurs in *rpl2* and *ycf1* genes. In this study, cp genome exhibited a loss of the *rpl32* gene in *D. zibethinus* (Fig. 4). This phenomenon may be attributed to abnormal DNA replication, repair, or recombination during the evolutionary process of their common ancestor [81, 82].

### Potential molecular markers in Malvaceae

Although the cp. genome evolves slowly and is relatively conserved, it still contains several mutational hotspots frequently used for species identification and molecular markers, such as *rbcL*, *ndhF*, *matK*, and *psbA-trnH* [76, 83]. These loci provide valuable genetic variation for phylogenetic studies and biodiversity assessments. The IR region is crucial for stabilizing the structure of the cp genome. Compared to the two single-copy regions, its slower nucleotide substitution rate enhances the efficacy of copy correction mechanisms [84]. Through the assembly of six cp. genomes, we found that the variability in the SSC and LSC regions is higher than that in the IR regions, and the variability in non-coding regions is higher than that in coding regions [11, 85].These findings are consistent with studies of cp genomes in other higher plants. Additionally, we identified seven highly variable regions, among which the *ycf1* gene shows potential for species identification. These results also indicate that the non-coding regions in the Malvaceae evolve at a faster rate than the coding regions. Therefore, when selecting molecular markers for species in the Malvaceae, non-coding regions should be prioritized.

Repetitive sequences can enhance genetic diversity within species and influence cpDNA rearrangements [40, 86]. Gene duplication serves as a significant source of organelle evolution and novel genetic functions [87]. Duplications in the single-copy regions are typically triggered by expansions of the IR regions. Overall, cpSSRs are valuable genetic tools for population genetics and evolutionary studies due to their codominance, high polymorphism, and low substitution rates [88, 89]. In our study, we identified a total of 50–96 SSRs across the six species (Fig. 3). SSRs are valuable genetic markers for distinguishing closely related species. In the six species we studied, mononucleotide SSRs were the most prevalent, aligning with observations in other Malvaceae species [4, 40]. These SSRs also hold potential for the development of lineage-specific cpSSR markers [90].

### Phylogenetic relationships in Malvaceae

Cp genome contains essential genes and is often used in biotechnology or phylogenetic research [17]. In the Malvadendrina clade, the phylogenetic relationship between Malvoideae and Bombacoideae was the first to be established [34]. Currently, the phylogenetic relationships within the Malvaceae family remain contentious. Most phylogenetic studies on Malvaceae suggest that Helicteroideae occupies a basal position within the Malvadendrina clade. Phylogenies based on single genes or a few genes often suffer from limited sample sizes, resulting in topological inconsistencies and low support values [35]. Cp genome data confirmed a close relationship among Brownlowioideae, Tilioideae, and Dombeyoideae, with Brownlowioideae represented by a single sequence. The analysis further demonstrated that Brownlowioideae is the sister group to the other two subfamilies [23]. In this study, we employed complete cp genome sequences to perform a comprehensive phylogenetic analysis of the relationships among nine subfamilies within the Malvaceae family. This analysis has refined and updated the phylogenetic positioning of the six Malvaceae species included in our research.

The entire Malvaceae family is primarily divided into two major clades, Byttneriina and Malvadendrina, consistent with previously reported phylogenetic relationships [24, 37, 91]. In the phylogenetic studies of the Malvaceae family, most research indicates that the subfamily Helicteroideae is situated at the base of the Malvadendrina clade. However, analyses based on individual cp. genes suggest that Dombeyoideae is the earliest-diverging basal group. Our study supports the positioning of the subfamily Helicteroideae at the base of the Malvadendrina clade (Fig. 8).

## Conclusion

This study employed Illumina sequencing data to assemble and annotate the cp genomes of six species within the Malvaceae family, offering novel insights into the evolutionary patterns of their cp genomes. Compared to other Malvaceae species, the cp genomes of these taxa demonstrate considerable conservation in terms of genome size, structural organization, and gene content, with the notable exception of a highly variable *ycf1* gene. The molecular data presented herein provide an invaluable resource for advancing the understanding of evolutionary processes within the Malvaceae family.

## Electronic supplementary material

Below is the link to the electronic supplementary material.

Supplementary Material 1

Supplementary Material 2

Supplementary Material 3

Supplementary Material 4

Supplementary Material 5

Supplementary Material 6

Supplementary Material 7

Supplementary Material 8

Supplementary Material 9

Supplementary Material 10

## Data Availability

The study of new sequencing data has been deposited in the NCBI (https://www.ncbi.nlm.nih.gov/), accession number is PRJNA1130351.

## Abbreviations

CP: Chloroplast
DNASP: DNA sequence polymorphism
IR: Inverted repeat
LSC: Large single copy
ML: Maximum likelihood
PI: Nucleotide diversity
SSC: Small single copy
SSR: Simple sequence repeat
