# Supplementary figures and images for: Comparative genomics and phylogenetic analysis of six Malvaceae species based on chloroplast genomes
Source: BMC Plant Biol. 2024 Dec 26;24:1245. doi: 10.1186/s12870-024-05974-w (PMC11670485; doi:10.1186/s12870-024-05974-w)

# Trans-splicing Genes

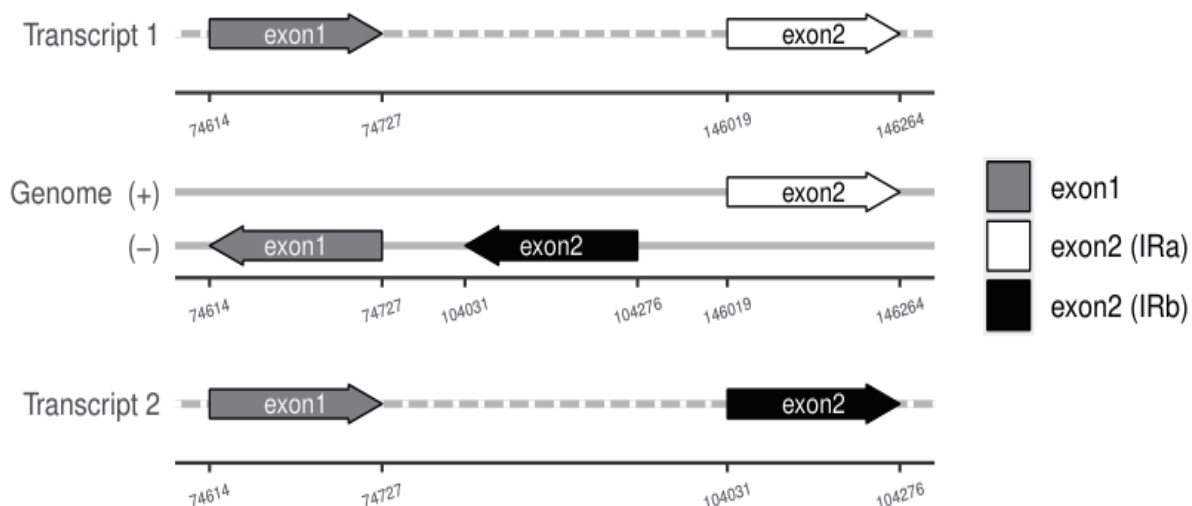

# Cis-splicing Genes

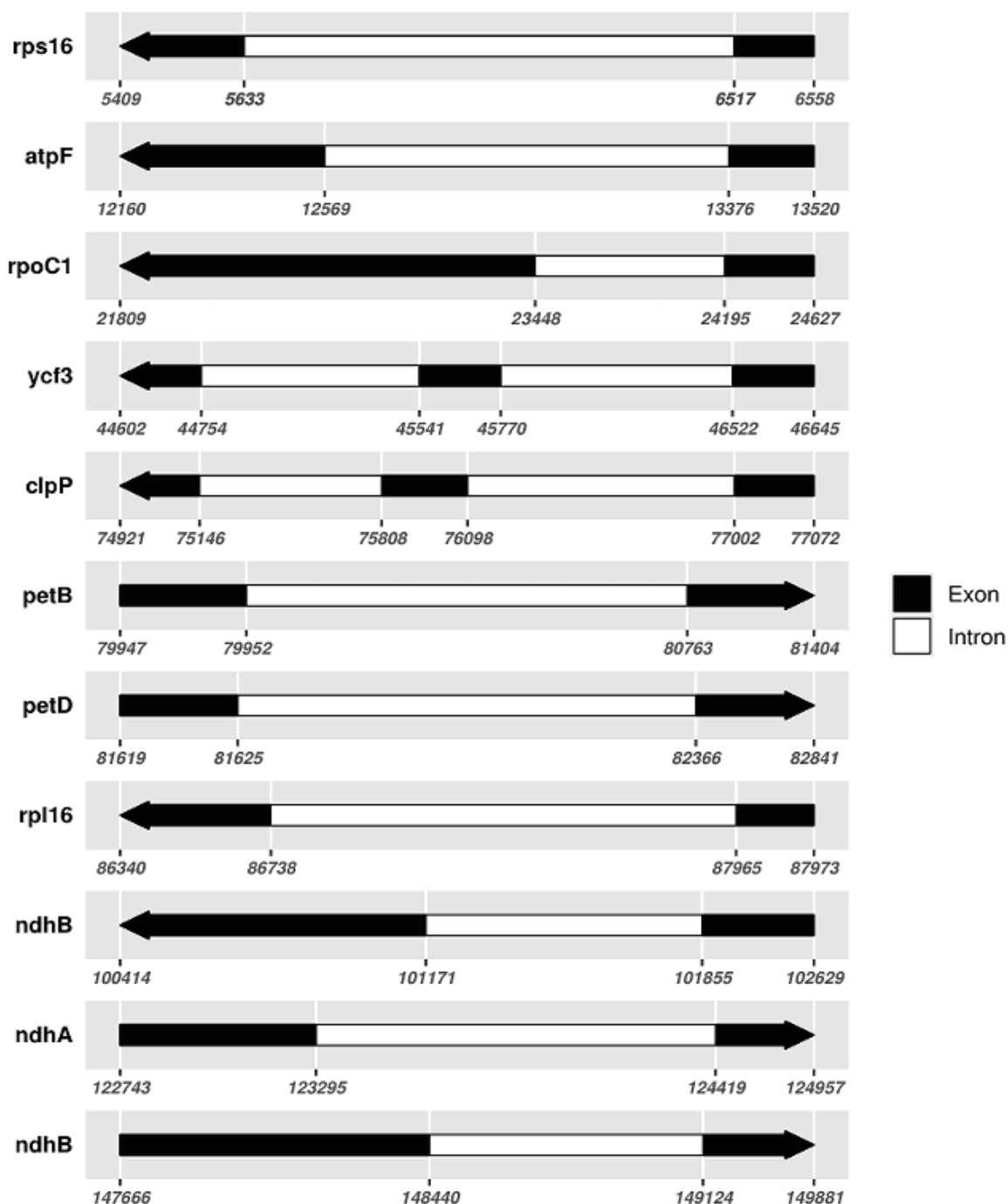

Supplement: Supplementary file 1 — Supplementary Material 1 [file 12870_2024_5974_MOESM1_ESM.pdf]

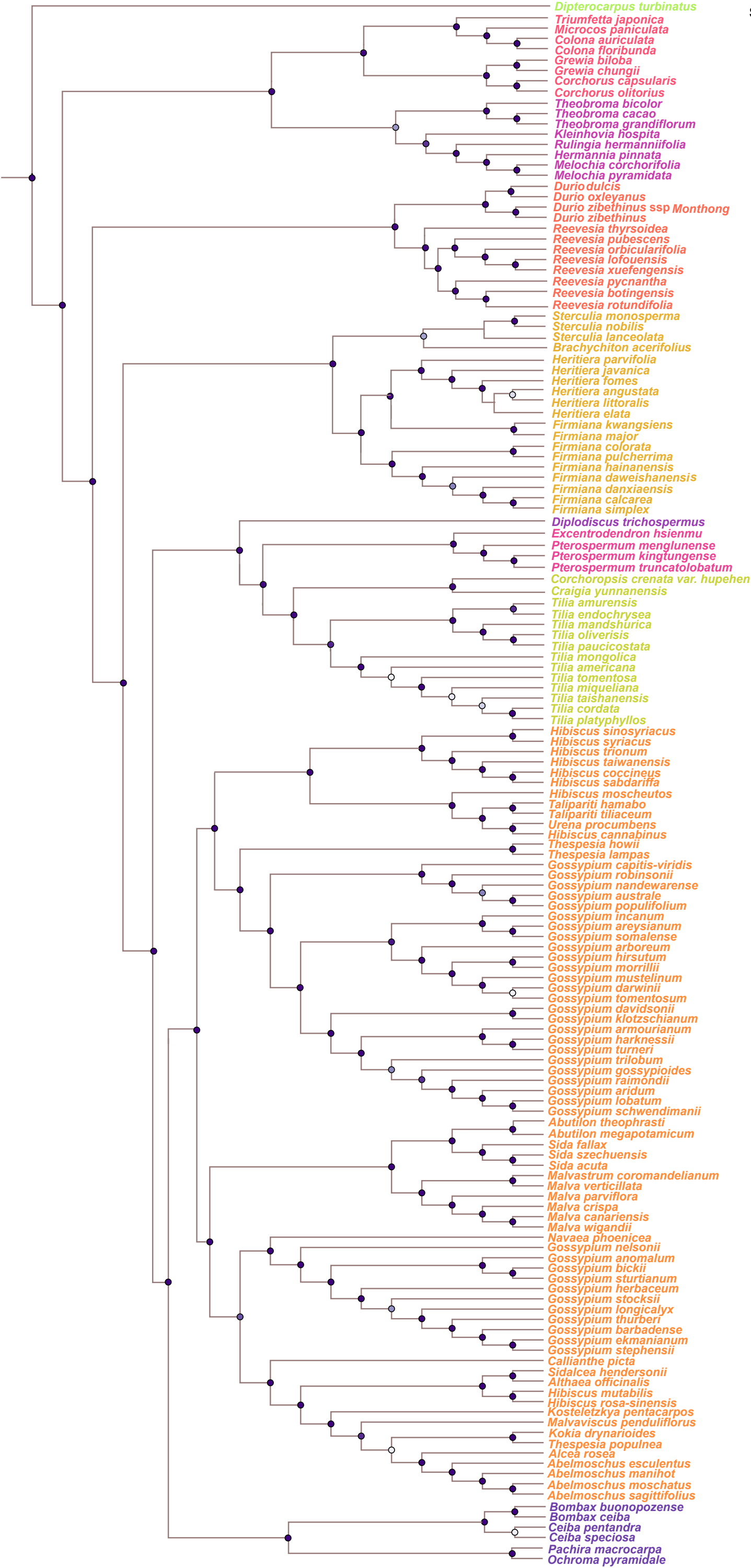

Supplement: Supplementary file 2 — Supplementary Material 2 [file 12870_2024_5974_MOESM2_ESM.pdf]
