# Supplementary material for: Comparative genomics and phylogenetic analysis of six Malvaceae species based on chloroplast genomes
Source: BMC Plant Biol. 2024 Dec 26;24:1245. doi: 10.1186/s12870-024-05974-w (PMC11670485; doi:10.1186/s12870-024-05974-w)
Supplement: Supplementary file 10 — Supplementary Material 10 [file 12870_2024_5974_MOESM10_ESM.docx]

**Table Legends**

**Table S1:** Overview of sequencing data for six Malvaceae species;

**Table S2:** The dataset of 141 Malvaceae cp genomes and 1 outgroup CP genome used in the study in public data;

**Table S3:** The number and ratio of amino acids encoded by each of the six Malvaceae species;

**Table S4:** The number and ratio of codon usage for each of the six Malvaceae species;

**Table S5:** Gene counts in the cp genomes of six Malvaceae species;

**Table S6:** Comparison of nucleotide polymorphism of six Malvaceae species;

**Table S7:** The dataset of 24 nuclear genomes and 1 outgroup used in the study in public data.

**Figure Legends**

**Fig. S1:** Exons and introns of cis-splicing genes and trans-splicing genes;

**Fig. S2:** Phylogenetic relationships among examined species with cp sequence of Malvaceae.
